# Association between socioeconomic status and physical inactivity in a general Japanese population: NIPPON DATA2010

**DOI:** 10.1371/journal.pone.0254706

**Published:** 2021-07-15

**Authors:** Yuka Sumimoto, Masahiko Yanagita, Naomi Miyamatsu, Nagako Okuda, Nobuo Nishi, Yosikazu Nakamura, Koshi Nakamura, Naoko Miyagawa, Motohiko Miyachi, Aya Kadota, Takayoshi Ohkubo, Tomonori Okamura, Hirotsugu Ueshima, Akira Okayama, Katsuyuki Miura, for NIPPON DATA2010 Research Group

**Affiliations:** 1 Department of Health and Sports Science, Doshisha University, Kyotanabe, Kyoto, Japan; 2 Department of Clinical Nursing, Shiga University of Medical Science, Tsukinowa-cho, Seta, Otsu, Shiga, Japan; 3 Department of Health Science, Kyoto Prefectural University, Sakyo-ku, Kyoto, Japan; 4 International Center for Nutrition and Information, National Institute of Health and Nutrition, National Institutes of Biomedical Innovation, Health and Nutrition, Shinjuku, Tokyo, Japan; 5 Department of Public Health, Jichi Medical University, Shimotsuke, Tochigi, Japan; 6 Department of Public Health and Hygiene, University of the Ryukyus, Nishihara, Okinawa, Japan; 7 Department of Preventive Medicine and Public Health, Keio University, Shinjuku, Tokyo, Japan; 8 Department of Physical Activity Research, National Institute of Health and Nutrition, National Institutes of Biomedical Innovation, Health and Nutrition, Shinjuku, Tokyo, Japan; 9 Department of Public Health, Shiga University of Medical Science, Tsukinowa-cho, Seta, Otsu, Shiga, Japan; 10 NCD Epidemiology Research Center, Shiga University of Medical Science, Tsukinowa-cho, Seta, Otsu, Shiga, Japan; 11 Department of Hygiene and Public Health, Teikyo University, Tokyo, Japan; 12 Research Institute of Strategy for Prevention, Tokyo, Japan; Ritsumeikan University, JAPAN

## Abstract

**Background:**

Lower socioeconomic status (SES) may be related to inactivity lifestyle; however, the association between SES and physical inactivity has not been sufficiently investigated in Japan.

**Methods:**

The study population is the participants of NIPPON DATA2010, which is a prospective cohort study of the National Health and Nutrition Survey 2010 in Japan. They were residents in 300 randomly selected areas across Japan. This study included 2,609 adults. Physical activity was assessed by physical activity index (PAI) calculated from activity intensity and time. The lowest tertile of PAI for each 10-year age class and sex was defined as physical inactivity. Multivariable logistic regression analyses were conducted to examine the association of SES (employment status, educational attainment, living status, and equivalent household expenditure (EHE)) with physical inactivity.

**Results:**

In the distribution of PAI by age classes and sex, the highest median PAI was aged 30–39 years among men (median 38.6), aged 40–49 years among women (38.0), and median PAI was decreased with increasing age. Multivariable-adjusted model shows that not working was significantly associated with physical inactivity after adjustment for age in all age groups and sexes. Not living with spouse for adult women and elderly men was significantly associated with physical inactivity compared to those who living with spouse. However, neither educational attainment nor EHE had any significant associations with physical inactivity.

**Conclusions:**

The result indicated that physical inactivity was associated with SES in a general Japanese population. SES of individuals need to be considered in order to prevent inactivity lifestyle.

## Introduction

Physical inactivity increases the risk of major non-communicable diseases (NCD), such as coronary heart disease, type 2 diabetes, and cancer (breast cancer and colon cancer), and shortened life expectancy [1]. The World Health Organization reported that physical inactivity is one of the primary four major risk factors leading for non-communicable diseases [2], and people who are insufficiently active have a 20% to 30% increased risk of death compared to people who are sufficiently active [3]. It is indicated that physical inactivity is related to low socioeconomic status (SES) [4].

In these last few decades, there is growing concern with the influence of SES; employment status, educational attainment, income level, etc., on health outcome. Previous studies have investigated the association between SES and physical inactivity, most of them from Western countries [5–8], and several studies from Japan [9–16], but the results have been inconsistent. There were some reasons for the inconsistent results of socioeconomic inequalities in physical activity. First, it might be caused by the contrasting for occupational activity and leisure time activity due to socioeconomic status [17]. Second, previous studies before 2010, occupation, the area of residence and age of the participants were limited [9–11], which may have caused selection bias in the participants, and there are concerns about the accuracy of the data because the data collection methods used were the Internet and mail questionnaires [12,13]. Since 2010, there have been several reports on the association between physical activity and mortality and/or disease risk factors [14,15], but only one study on the association between physical activity and socioeconomic status [16]. In recently, there are growing concerns that social inequalities may grow wider in Japan and generate a harmful effect on health [18].

Therefore, we examined to the association between SES and physical inactivity to identify subgroup with physical inactivity using the baseline data of NIPPON DATA2010. This cohort study is unique because the participants were general Japanese adult population from 300 randomly selected districts throughout Japan and focus on the impact of socioeconomic status on health [19]. Analysis of the causes of physical inactivity, specifically the association between SES and physical inactivity using the baseline data of NIPPON DATA2010 can provide insights into opportunities and priorities for prevention, intervention and policy to physical inactivity.

## Material and methods

### Study population

A prospective cohort study on cardiovascular disease, the National Integrated Project for Prospective Observation of Non-communicable Disease And its Trends in the Aged 2010 (NIPPON DATA2010) was established in 2010 [19]. This study was performed using data from the National Health and Nutrition Survey in November 2010 (NHNS2010) and the Comprehensive Survey of Living Conditions in June 2010 (CSLS2010), which were conducted by the Ministry of Health, Labour and Welfare of Japan.

In November 2010, 8,815 residents aged 1 year and older from 300 randomly selected districts throughout Japan participated in the dietary survey for NHNS2010. Among 7,229 participants age 20 years and older, 3,873 participants (1,598 men and 2,275 women) had a blood test of NHNS2010 and were invited to enroll in NIPPON DATA2010. A total of 2,898 participants (1,239 men and 1,659 women; participant rate, 74.6%) agreed to participate in the baseline survey for NIPPON DATA2010. Trained interviewers obtained written informed consent from all participants before enrollment. Data obtained from NHNS2010 and CSLC2010 were merged with data from NIPPON DATA2010. The Institutional Review Board of Shiga University of Medical Science (No. 22–29, 2010) approved this study.

For this study, of the 2,898 participants, 91 were excluded because it was not possible to merge data from NHNS2010 or CSLC2010 with NIPPON DATA2010 baseline data. Additionally, seven who were over 90 years old, 150 who could not exercise due to health reasons, and 41 who were lacking main variables were excluded. The remaining 2,609 participants (1,132 men and 1,477 women) were included in the present study.

### Physical activity index

To evaluate physical activity, questions were posed about number of hours per day spent in the baseline survey of NIPPON DATA2010; the interviewer ensured that the total time added up to 24h. Physical activity by intensity was defined as follows; (1) heavy activity (construction work, agriculture, sports such as jogging, etc); (2) moderate activity (light work done standing, housework, gardening and walking, etc); (3) slight activity (light work done sitting, office work, driving a car, eating and taking a bath, etc); (4) watching television (TV) and other sedentary (sitting such as reading); (5) no activity (sleeping and lying down).

Physical activity index (PAI) was calculated by multiplying the time spent in different activities by corresponding weighting factors that parallel the increased rate of oxygen consumption associated with increasingly more intense physical activity (weighting factors; heavy activity for 5.0, moderate activity for 2.4, slight activity for 1.5, watching TV and other sedentary for 1.1 and no activity for 1.0); the procedure used in the Framingham Offspring study was followed [20].

Total physical activity index = 5.0×hours of heavy activity + 2.4×hours of moderate activity + 1.5×hours of slight activity + 1.1×hours of watching TV and other sedentary + 1.0× hours of no activity.

It was not possible to determine a cutoff value that defines a state in which the amount of moderate-vigorous intensity physical activity performed per week is less than the recommended amount, as in the WHO guidelines, because PAI was calculated from daily physical activity. Therefore, we referred to that previous studies which divided PAI into tertiles and classified the lowest tertile as insufficient physical activity in the Framingham Study [21,22]. In this study, we confirmed the distribution of the physical activity index and found that it differed greatly by sex and age. To account for these differences, we divided the PAI by sex and age class and defined the lowest tertile for each as physically inactive.

### Socioeconomic status

Information on SES was collected using self-administered questionnaires for NHNS2010 (employment status), CSLC2010 (living status, monthly household expenditure of May 2010, number of family member, house ownership) and NIPPON DATA2010 (educational attainment). Equivalent household expenditure (EHE) were calculated as monthly household expenditure divided by the square root of the number of family member and categorized into tertile. House ownership was used to adjust the EHE, because in the CSLC questionnaire, rent in non-house owners was taken into account as a part of expenditure, but mortgate payments in home owner was not.

SES was defined as follow: (1) employment status (working [including self-employed] or not working [including students and homemakers]); (2) educational attainment (junior high school, high school, college or higher); (3) living status (living with spouse or not living with spouse); (4) EHE (first tertile [less than 106,000 yen], second tertile [106,000 yen or more but less than 162,000 yen], third tertile [162,000 yen or more]).

### Lifestyle and other variables

Public health nurses collected information on alcohol drinking habit, smoking habit and past histories of myocardial infarction and stroke using a standardized questionnaire in NHNS. Alcohol drinking habit, smoking habit and past histories were obtained from NHNS2010. Participants had past histories of myocardial infarction and/or stroke were defined as having past histories. These were classified as follow: (1) alcohol drinking habit (current drinker, ex-drinker or non-drinker); (2) smoking habit (current smoker, ex-smoker or non-smoker); (3) Past histories (yes or no).

### Statistical analysis

Statistical analyses were performed for men/women and for adult (aged 20–59 years)/elderly (aged 60–89 years), separately, because basic living practice, e.g., working or not working, would differ substantially by sex and by age groups. In addition, the age of retirement was usually set at 60 years of age for indefinite-term employees at most workplaces in Japan.

To evaluate physical inactivity in each age class, PAI was divided into tertiles in seven age classes (20–29, 30–39, 40–49, 50–59, 60–69, 70–79, and 80–89) for men and women separately.

To evaluate association of SES and physical inactivity, the odds ratios (ORs) and 95% confidence intervals (95% CIs) for physical inactivity were calculated by multiple logistic regression analyses, using explanatory variable (employment status, educational attainment, living status and EHE) and possible confounding factors (alcohol drinking habit, smoking habit and past histories). We used three models. Model l was adjusted for age. Model 2 was further adjusted with alcohol drinking habit, smoking habit and past histories. For Model 3, we put all the SES factors and confounding factors simultaneously. For analyses on EHE, we additionally adjusted for house ownership (owned or rented). P<0.05 was considered statically significant. All statistical analyses were performed using SPSS version 24 for Windows.

## Results

### Characteristics of participants

Table 1 shows the distribution of age, employment status, educational attainment, EHE, and other variable by sex and age groups. For employment status, adult participants who were not working were 6.1% for men and 35.3% for women. Elderly participants who were not working were 55.0% for men and 76.6% for women. For educational attainment, approximately half of adult participants graduated from college or higher (48.1% for men and 51.7% for women), whereas most elderly participants of both sexes graduated from high school or junior high school. For living status, most adult women lived with spouse (84.6%), whereas the rate in elderly women was lower (65.5%).

**Table 1:** Characteristics of study participants by sex and age groups, NIPPON DATA2010, 2010, Japan.

	Men (*n* = 1,132)					Women (*n* = 1,477)			
	Adult			Elderly		Adult		Elderly	
	(20–59 years)			(60–89 years)		(20–59 years)		(60–89 years)	
*N* (%)	457	(40.4)		675	(59.6)	720	(48.7)	757	(51.3)
Age, years (SD)	44.1	(10.7)		70.1	(6.9)	43.8	(10.4)	70.1	(6.9)
Body mass index, kg/m^2^ (SD)	24.1	(3.6)		23.8	(2.8)	21.9	(3.5)	23.1	(3.4)
Employment status, *n* (%)									
Working	429	(93.9)		304	(45.0)	466	(64.7)	177	(23.4)
Not working	28	(6.1)		371	(55.0)	254	(35.3)	580	(76.6)
Educational attainment, *n* (%)									
Junior high school	32	(7.0)		247	(36.6)	50	(6.9)	288	(38.0)
High school	205	(44.9)		276	(40.9)	298	(41.4)	373	(49.3)
College or higher	220	(48.1)		152	(22.5)	372	(51.7)	96	(12.7)
Living status, *n* (%)									
Living with spouse	330	(72.2)		571	(84.6)	547	(76.0)	496	(65.5)
Not living with spouse	127	(28.8)		104	(15.4)	173	(24.0)	261	(34.5)
Equivalent household expenditure, *n* (%)									
1st tertile	167	(36.5)		193	(28.6)	205	(28.5)	248	(32.8)
2nd tertile	155	(33.9)		252	(37.3)	264	(36.7)	262	(34.6)
3rd tertile	135	(29.5)		230	(34.1)	251	(34.9)	247	(32.6)
Smoking habit, *n* (%)									
Current smoker	176	(38.5)		136	(20.1)	79	(11.0)	16	(2.1)
Ex-smoker	125	(27.4)		303	(44.9)	61.0	(8.5)	29	(3.8)
Non-smoker	156	(34.1)		236	(35.0)	580	(80.6)	712	(94.1)
Alcohol drinking habit, *n* (%)									
Current drinker	339	(74.2)		488	(72.3)	344	(47.8)	198	(26.2)
Ex-drinker	6	(1.3)		30	(4.4)	12	(1.7)	8	(1.1)
Non-drinker	112	(24.5)		157	(23.3)	364	(50.6)	551	(72.8)
Exercise habits, *n* (%)									
Exercise	119	(74.0)		324	(48.0)	168	(23.3)	328	(43.3)
Not have exercise habits	338	(26.0)		351	(52.0)	552	(76.7)	429	(56.7)
House ownership, *n* (%)									
Own house	344	(75.3)		574	(85.0)	540	(75.0)	661	(87.3)
Rented house	113	(24.7)		101	(15.0)	180	(25.0)	96	(12.7)
Number of household menber, *n* (%)									
One	53	(11.6)		85	(12.6)	41	(5.7)	167	(22.1)
Two	78	(17.1)		347	(51.4)	170	(23.6)	350	(46.2)
Three or over	326	(71.3)		243	(36.0)	509	(70.7)	240	(31.7)
Past histories, *n* (%)									
Myocardial infarction	3	(0.7)		30	(4.4)	0	(0.0)	11	(1.5)
Stroke	8	(1.8)		48	(7.1)	1	(0.1)	33	(4.4)
Any of them	10	(2.2)		73	(10.8)	1	(0.1)	42	(5.5)

SD; standard deviation.

Data are presented as mean (SD) or as a number (%).

### The distribution of physical activity index

Table 2 shows median and interquartile range of PAI for men and women in seven age classes. Among the seven age classes, median of total PAI, interquartile range was highest in aged 30–39 years among men (median 38.6), while it was highest in aged 40–49 years among women (38.0). The median of total PAI decreased with increasing age, and aged 80–89 years was the lowest in both men (30.8) and women (32.9). Regarding median PAI by each intensity of activities in sex and age classes, PAI of moderate activity included housework was higher score than PAI of other activity in women (S1 Table).

**Table 2:** Distribution of physical activity index by sex and age classes.

			Total		1st tertile		2nd tertile		3rd tertile	
	*n*	(%)	Median	(IQR)	Median	(IQR)	Median	(IQR)	Median	(IQR)
**Men**										
20–29 years	52	(4.6)	38.5	(31.3,41.2)	30.6	(29.4,31.7)	38.7	(36.9,39.2)	43.1	(41.1,53.0)
30–39 years	103	(9.1)	38.6	(31.7,43.1)	31.3	(30.4,31.7)	38.6	(35.4,39.8)	56.9	(43.1,62.0)
40–49 years	122	(10.8)	37.6	(31.4,44.2)	30.5	(30.1,31.4)	37.5	(32.9,39.9)	59.7	(44.0,65.8)
50–59 years	180	(15.9)	36.1	(31.2,41.2)	30.5	(30.0,31.2)	36.1	(33.9,38.0)	51.0	(41.3,62.4)
60–69 years	345	(30.9)	35.3	(30.7,40.1)	29.4	(28.1,30.5)	35.2	(32.6,37.0)	47.1	(40.0,58.0)
70–79 years	252	(22.3)	32.4	(29.6,39.4)	28.0	(27.1,29.6)	32.4	(31.6,34.8)	42.5	(39.4,52.2)
80–89 years	78	(6.9)	30.8	(27.9,35.2)	27.4	(26.8,27.9)	30.8	(29.7,32.6)	37.2	(35.3,48.2)
**Women**										
20–29 years	64	(4.3)	35.7	(31.4,39.3)	30.6	(29.1,31.4)	35.1	(33.5,37.0)	40.5	(38.8,43.3)
30–39 years	221	(15.0)	37.7	(33.6,40.9)	32.0	(30.7,33.7)	37.7	(36.7,38.7)	41.9	(40.7,44.6)
40–49 years	174	(11.8)	38.0	(34.4,41.3)	33.1	(31.6,34.5)	38.0	(37.1,39.3)	43.0	(41.2,46.1)
50–59 years	261	(17.7)	37.4	(33.9,40.9)	32.2	(30.9,33.9)	37.4	(36.5,38.2)	42.3	(40.8,44.6)
60–69 years	389	(26.3)	36.4	(33.6,40.4)	32.4	(31.1,33.7)	36.4	(35.6,37.6)	42.0	(40.5,44.9)
70–79 years	283	(19.2)	35.9	(32.3,39.4)	31.4	(29.2,32.2)	35.9	(34.5,37.1)	41.7	(39.4,47.4)
80–89 years	85	(5.8)	32.9	(29.6,36.7)	28.6	(26.7,29.5)	32.7	(31.9,33.5)	39.3	(36.2,45.6)

IQR; Inter-Quartile Range.

### Association between socioeconomic status and physical inactivity

Tables 3 and 4 shows results from multiple logistic regression analysis using physical inactivity as an objective variable and socioeconomic factors as explanatory variables. Results were almost similar in three models. In Model 3, significantly increased ORs for physical inactivity were observed for not working participants compared with working participants after adjustment age in all strata (OR 3.38 in adult men, 1.46 in adult women, 2.17 in elderly men, 1.72 in elderly women). For living status, there was no significant association with physical inactivity in adult men. However, elderly men not living with spouse had higher OR for physical inactivity than those who were living with spouse (OR 2.01). On the other hand, adult women not living with spouse had higher OR for physical inactivity than those who were living with spouse (OR 1.63), although there was no significant association in elderly women. Regarding educational attainment and EHE, neither of them showed any significant associations with physical inactivity in all strata. Results were similar even after adjusting for body mass index and living with others (S2 Table).

**Table 3:** Association between socioeconomic status and physical inactivity in men (*n* = 1,132).

			Model 1		Model 2		Model 3	
	*n*	%^a^	OR	95% CI	OR	95% CI	OR	95% CI
**Adult(20–59 years)**								
Employment status								
Working	429	31.9		(ref.)		(ref.)		(ref.)
Not working	28	53.6	**2.46**	**(1.14−5.32)**	**2.84**	**(1.26−6.38)**	**3.38**	**(1.43−7.99)**
Educational attainment								
Junior high school	32	28.1		(ref.)		(ref.)		(ref.)
High school	205	26.3	0.91	(0.39−2.09)	0.95	(0.41−2.22)	0.90	(0.37−2.20)
College or higher	220	40.5	1.71	(0.75−3.93)	1.88	(0.80−4.40)	1.83	(0.75−4.49)
Living status								
Living with spouse	330	33.3		(ref.)		(ref.)		(ref.)
Not living with spouse	127	33.1	0.94	(0.59−1.49)	0.93	(0.58−1.48)	0.81	(0.49−1.34)
Equivalent household expenditure								
1st tertile	167	29.9		(ref.)		(ref.)		(ref.)
2nd tertile	155	32.3	1.13	(0.70−1.82)	1.17	(0.72−1.89)	1.14	(0.70−1.87)
3rd tertile	135	38.5	1.48	(0.91−2.39)	1.51	(0.93−2.46)	1.36	(0.83−2.25)
**Elderly (60–89 years)**								
Employment status								
Working	304	23.0		(ref.)		(ref.)		(ref.)
Not working	371	40.4	**2.32**	**(1.61**−**3.32)**	**2.31**	**(1.61−3.31)**	**2.17**	**(1.51−3.14)**
Educational attainment								
Junior high school	247	31.6		(ref.)		(ref.)		(ref.)
High school	276	31.2	1.03	(0.71−1.50)	1.05	(0.72−1.53)	0.98	(0.66−1.44)
College or higher	152	36.8	1.31	(0.85−2.01)	1.34	(0.87−2.06)	1.22	(0.77−1.92)
Living status								
Living with spouse	571	30.6		(ref.)		(ref.)		(ref.)
Not living with spouse	104	43.3	**1.74**	**(1.13−2.67)**	**1.73**	**(1.12−2.65)**	**1.63**	**(1.03−2.56)**
Equivalent household expenditure								
1st tertile	193	27.5		(ref.)		(ref.)		(ref.)
2nd tertile	252	35.7	1.46	(0.97−2.20)	1.49	(0.99−2.25)	1.43	(0.94−2.19)
3rd tertile	230	33.5	1.33	(0.87−2.02)	1.38	(0.90−2.10)	1.28	(0.82−1.99)

OR, odds ratio; CI, confidence intervals.

^a^Propotion of defined as participants who physical inactiviy.Physical activity index (PAI) was divided tertile by sex for each 10-year age category and the lowest tertile was defined as physical inactivity. Mode l was adjusted for age (additionaly adjusted for house ownership for equivalent household expenditure). Model 2 was adjusted for variables in model 1 plus past histories, alcohol drinking habit and smoking habit. Model 3 was adjusted for variables in model 2, simultaneously.

**Table 4:** Association between socioeconomic status and physical inactivity in women (*n* = 1,477).

			Model 1		Model 2		Model 3	
	*n*	%^a^	OR	95% CI	OR	95% CI	OR	95% CI
**Adult(20–59 years)**								
Employment status								
Working	466	31.3		(ref.)		(ref.)		(ref.)
Not working	254	37.0	**1.29**	**(0.93**−**1.78)**	**1.29**	**(0.93**−**1.78)**	**1.46**	**(1.04**−**2.04)**
Educational attainment								
Junior high school	50	42.0		(ref.)		(ref.)		(ref.)
High school	298	31.9	0.65	(0.35−1.19)	0.63	(0.34−1.17)	0.63	(0.33−1.20)
College or higher	372	33.3	0.69	(0.38−1.27)	0.66	(0.36−1.24)	0.66	(0.35−1.26)
Living status								
Living with spouse	547	30.2		(ref.)		(ref.)		(ref.)
Not living with spouse	173	43.4	**1.86**	**(1.29**−**2.68)**	**1.86**	**(1.29**−**2.69)**	**2.01**	**(1.37**−**2.94)**
Equivalent household expenditure								
1st tertile	205	29.8		(ref.)		(ref.)		(ref.)
2nd tertile	264	36.7	1.36	(0.92−2.02)	1.36	(0.92−2.02)	1.42	(0.95−2.12)
3rd tertile	251	32.7	1.15	(0.77−1.72)	1.14	(0.76−1.71)	1.13	(0.75−1.71)
**Elderly (60–89 years)**								
Employment status								
Working	177	25.4		(ref.)		(ref.)		(ref.)
Not working	580	35.0	**1.60**	**(1.08**−**2.36)**	**1.63**	**(1.10**−**2.42)**	**1.72**	**(1.15**−**2.57)**
Educational attainment								
Junior high school	288	32.6		(ref.)		(ref.)		(ref.)
High school	373	33.1	1.04	(0.75−1.45)	1.05	(0.75−1.47)	1.09	(0.78−1.54)
College or higher	96	31.3	0.95	(0.58−1.58)	1.02	(0.62−1.70)	1.12	(0.66−1.89)
Living status								
Living with spouse	496	30.6		(ref.)		(ref.)		(ref.)
Not living with spouse	261	36.8	1.33	(0.95−1.87)	1.32	(0.94−1.85)	1.31	(0.92−1.87)
Equivalent household expenditure								
1st tertile	248	33.5		(ref.)		(ref.)		(ref.)
2nd tertile	262	31.3	0.90	(0.62−1.31)	0.90	(0.62−1.31)	0.83	(0.57−1.23)
3rd tertile	247	33.6	1.01	(0.70−1.47)	1.03	(0.71−1.50)	0.97	(0.65−1.43)

OR, odds ratio; CI, confidence intervals.

^a^Propotion of defined as participants who physical inactiviy.Physical activity index (PAI) was divided tertile by sex for each 10-year age category and the lowest tertile was defined as physical inactivity. Mode l was adjusted for age (additionaly adjusted for house ownership for equivalent household expenditure). Model 2 was adjusted for variables in model 1 plus past histories, alcohol drinking habit and smoking habit. Model 3 was adjusted for variables in model 2, simultaneously.

## Discussion

In the present analysis of a nationwide cross-sectional study of a randomly selected sample of adults in a Japanese population, it was indicated that the detailed distribution of PAI by age classes and sex, and the association between SES and physical inactivity in a general Japanese population. A main finding was that not working was related to physical inactivity in all strata. In addition, not living with spouse for adult women and elderly men was related to physical inactivity compared with living with spouse.

Regarding employment status, numerous previous studies reported that not working persons were physically inactive compared to those who were working [8,23–25]. This study in Japan supports the result of previous studies, and significant association was found not only in adult but also in elderly. The reason why not working elderly had a significantly associated with physical inactivity, it is possible that lifestyle habits changed. According to previous studies, the elderly had prolonged television viewing time and sedentary time after retirement [26–28]. These phenomena may be partly explained that not working elderly was likely to be physically inactive in this study.

Regarding living status, the results of previous studies were not consistent [23,29–31]. In this study, adult women and elderly men not living with spouse had a significantly higher risk of physical inactivity. The reason for physical inactivity in adult women not living with spouse may be related to the gender difference in housework time due to marital status. In this study, 71.3% of adult women not living with spouse were unmarried. According to a survey by the Cabinet Office of Japan, between unmarried and married women, there was a slight difference in working time, but there was 4-hour difference in housework time; on the other hand, in men, there was little difference in working time and housework time due to marital status [32]. As mentioned above, the gender role difference due to marital status may account for physical inactivity in adult women in Japan.

The reason for physical inactivity in elderly men not living with spouse may be related to the gender difference due to effects from being widowed or divorced. In fact, previous study reported that 80% of married men recognized their spouse as a person who controlled for their health [33]. Some studies reported that elderly men after widow or divorce decreased vegetable intake, increased smoking and alcohol consumption, and had higher stress and depression [34–37]. On the other hand, no association was found in elderly women. According to a survey by the Cabinet Office of Japan, approximately 80% of elderly men responded that their spouses give “mental support,” whereas only a half of women responded so [38]. The gender difference in support by spouse may account for the difference in the relationship of spouse to physical inactivity.

From the reason above, widowed or divorced men may be particularly physically inactive. A previous study showed that loneliness was an independent risk factor for physical inactivity in the elderly [39]. Loneliness caused by loss of occupation, social status due to retirement, and loss of mental support due to widow or divorce may be related to physical inactivity. Therefore, avoiding loneliness would be useful to prevent physical inactivity. Several studies reported that there was a positive association between neighborhood relationship and moderate to vigorous physical activity [40,41].

In this study, education attainment and EHE were not any significantly associated with physical inactivity. The results were somewhat inconsistent with most previous studies in which educational attainment and income were one of determinants of physical inactivity [5,6,31]. Compared to other countries, our results suggest that the impact of socioeconomic disparities according to educational attainment and income to physical inactivity may be small. Therefore, to identify subgroup which need intervention to prevent physical inactivity in Japan, we should be focus on employment status and living status.

This study has several limitations. First, because of the cross-sectional nature of this study, we were unable to determine whether there was a casual association between SES and physical inactivity. The second, we investigated total physical activity hours which were classified by intensity; thus, the type of physical activity could not be assessed.

Finally, physical activity was assessed using self-administered questionnaires, recall bias may have occurred, and physical activity may have been overestimated or underestimated.

## Conclusion

The present study from a nationwide survey of the general Japanese population demonstrated that in the distribution of PAI, the highest median PAI was differed by age and sex, however PAI decreased with increasing age in both sexes. In the association between SES and physical inactivity, not working was associated with physical inactivity regardless of age and sex, whereas not living with spouse was differently associated by age and sex. These results will contribute to public health interventions which prevent socioeconomic inequalities in physical inactivity.

## Supporting information

S1 Table Distribution of physical activity index by each intensity of activities. (PDF)

S2 Table Association between socioeconomic status and physical inactivity. (PDF)

## References

[1] Lee IM, Shiroma EJ, Lobelo F, Puska P, Blair SN, Katzmarzyk PT; Lancet Physical Activity Series Working Group. Effect of physical inactivity on major non-communicable diseases worldwide: an analysis of burden of disease and life expectancy. Lancet. 2012 Jul 21;380(9838):219–29. doi: 10.1016/S0140-6736(12)61031-9 22818936 PMC3645500

[2] World Health Organization. Noncommunicable diseases [Internet]. 2021. [Cited 2021 May 28] Available from: https://www.who.int/health-topics/noncommunicable-diseases#tab=tab_1.

[3] World Health Organization. Physical activity [Internet]. [Cited 2021 May 28] Available from: https://www.who.int/news-room/fact-sheets/detail/physical-activity.

[4] World Health Organization. Social Determinants of Health, The Solid Facts, second edition. Geneva, 2003. [Cited 2021 May 28] Available from: https://www.euro.who.int/__data/assets/pdf_file/0005/98438/e81384.pdf.

[5] Crespo CJ, Ainsworth BE, Keteyian SJ, Heath GW, Smit E. Prevalence of physical inactivity and its relation to social class in U.S. adults: results from the Third National Health and Nutrition Examination Survey, 1988–1994. Med Sci Sports Exerc. 1999 Dec;31(12):1821–7. doi: 10.1097/00005768-199912000-00019 10613434

[6] Norman A, Bellocco R, Vaida F, Wolk A. Total physical activity in relation to age, body mass, health and other factors in a cohort of Swedish men. Int J Obes Relat Metab Disord. 2002 May;26(5):670–5. doi: 10.1038/sj.ijo.0801955 12032752

[7] Carlson SA, Densmore D, Fulton JE, Yore MM, Kohl HW 3rd. Differences in physical activity prevalence and trends from 3 U.S. surveillance systems: NHIS, NHANES, and BRFSS. J Phys Act Health. 2009;6 Suppl 1:S18–27. doi: 10.1123/jpah.6.s1.s18 19998846

[8] Van Domelen DR, Koster A, Caserotti P, Brychta RJ, Chen KY, McClain JJ, et al. Employment and physical activity in the U.S. Am J Prev Med. 2011 Aug;41(2):136–45. doi: 10.1016/j.amepre.2011.03.019 21767720 PMC5221416

[9] Nishi N, Makino K, Fukuda H, Tatara K. Effects of socioeconomic indicators on coronary risk factors, self-rated health and psychological well-being among urban Japanese civil servants. Soc Sci Med. 2004 Mar;58(6):1159–70. doi: 10.1016/s0277-9536(03)00287-9 14723910

[10] Takao S, Kawakami N, Ohtsu T; Japan Work Stress and Health Cohort Study Group. Occupational class and physical activity among Japanese employees. Soc Sci Med. 2003 Dec;57(12):2281–9. doi: 10.1016/s0277-9536(03)00134-5 14572837

[11] Fukuda Y, Nakamura K, Takano T. Accumulation of health risk behaviours is associated with lower socioeconomic status and women’s urban residence: a multilevel analysis in Japan. BMC Public Health. 2005 May 27;5:53. doi: 10.1186/1471-2458-5-53 15921512 PMC1174875

[12] Shibata A, Oka K, Nakamura Y, Muraoka I. Prevalence and demographic correlates of meeting the physical activity recommendation among Japanese adults. J Phys Act Health. 2009 Jan;6(1):24–32. doi: 10.1123/jpah.6.1.24 19211955

[13] Liao Y, Harada K, Shibata A, Ishii K, Oka K, Nakamura Y, et al. Association of self-reported physical activity patterns and socio-demographic factors among normal-weight and overweight Japanese men. BMC Public Health. 2012 May 21;12:278. doi: 10.1186/1471-2458-12-278 22490124 PMC3357319

[14] Sakaue A, Adachi H, Enomoto M, Fukami A, Kumagai E, Nakamura S, et al. Association between physical activity, occupational sitting time and mortality in a general population: An 18-year prospective survey in Tanushimaru, Japan. Eur J Prev Cardiol. 2020 May;27(7):758–766. doi: 10.1177/2047487318810020 30396293

[15] Kikuchi H, Inoue S, Lee IM, Odagiri Y, Sawada N, Inoue M, et al. Impact of Moderate-Intensity and Vigorous-Intensity Physical Activity on Mortality. Med Sci Sports Exerc. 2018 Apr;50(4):715–721. doi: 10.1249/MSS.0000000000001463 29053480

[16] Matsushita M, Harada K, Arao T. Socioeconomic position and work, travel, and recreation-related physical activity in Japanese adults: a cross-sectional study. BMC Public Health. 2015 Sep 18;15:916. doi: 10.1186/s12889-015-2226-z 26385476 PMC4575444

[17] Beenackers MA, Kamphuis CB, Giskes K, Brug J, Kunst AE, Burdorf A, et al. Socioeconomic inequalities in occupational, leisure-time, and transport related physical activity among European adults: a systematic review. Int J Behav Nutr Phys Act. 2012 Sep 19;9:116. doi: 10.1186/1479-5868-9-116 22992350 PMC3491027

[18] Kagamimori S, Gaina A, Nasermoaddeli A. Socioeconomic status and health in the Japanese population. Soc Sci Med. 2009 Jun;68(12):2152–60. doi: 10.1016/j.socscimed.2009.03.030 Epub 2009 Apr 16. 19375838

[19] Kadota A, Okuda N, Ohkubo T, Okamura T, Nishi N, Ueshima H, et al. The National Integrated Project for Prospective Observation of Non-communicable Disease and its Trends in the Aged 2010 (NIPPON DATA2010): Objectives, Design, and Population Characteristics. J Epidemiol. 2018;28 Suppl 3 (Suppl 3):S2–S9. doi: 10.2188/jea.JE20170240 29503381 PMC5825689

[20] Kannel WB, Sorlie P. Some health benefits of physical activity. The Framingham Study. Arch Intern Med. 1979 Aug;139(8):857–61. 464698

[21] Jonker JT, De Laet C, Franco OH, Peeters A, Mackenbach J, Nusselder WJ. Physical activity and life expectancy with and without diabetes: life table analysis of the Framingham Heart Study. Diabetes Care. 2006 Jan;29(1):38–43. doi: 10.2337/diacare.29.01.06.dc05-0985 16373893

[22] Ballard-Barbash R, Schatzkin A, Albanes D, Schiffman MH, Kreger BE, Kannel WB, et al. Physical activity and risk of large bowel cancer in the Framingham Study. Cancer Res. 1990 Jun 15;50(12):3610–3. . 2340509

[23] Al-Tannir M, Kobrosly S, Itani T, El-Rajab M, Tannir S. Prevalence of physical activity among Lebanese adults: a cross-sectional study. J Phys Act Health. 2009 May;6(3):315–20. doi: 10.1123/jpah.6.3.315 19564659

[24] Ying C, Kuay LK, Huey TC, Hock LK, Hamid HA, Omar MA, et al. Prevalence and factors associated with physical inactivity among Malaysian adults. Southeast Asian J Trop Med Public Health. 2014 Mar;45(2):467–80. 24968689

[25] Newtonraj A, Murugan N, Singh Z, Chauhan RC, Velavan A, Mani M. Factors Associated with Physical Inactivity among Adult Urban Population of Puducherry, India: A Population Based Cross-sectional Study. J Clin Diagn Res. 2017 May;11(5):LC15–LC17. doi: 10.7860/JCDR/2017/24028.9853 Epub 2017 May 1. 28658812 PMC5483714

[26] Touvier M, Bertrais S, Charreire H, Vergnaud AC, Hercberg S, Oppert JM. Changes in leisure-time physical activity and sedentary behaviour at retirement: a prospective study in middle-aged French subjects. Int J Behav Nutr Phys Act. 2010 Feb 4;7:14. doi: 10.1186/1479-5868-7-14 20181088 PMC2834610

[27] Suorsa K, Pulakka A, Leskinen T, Heinonen I, Heinonen OJ, Pentti J, et al. Objectively Measured Sedentary Time Before and After Transition to Retirement: The Finnish Retirement and Aging Study. J Gerontol A Biol Sci Med Sci. 2020 Sep 16;75(9):1737–1743. doi: 10.1093/gerona/glz127 31095675

[28] Yonemoto K, Honda T, Kishimoto H, Yoshida D, Hata J, Mukai N, et al. Longitudinal Changes of Physical Activity and Sedentary Time in the Middle-Aged and Older Japanese Population: The Hisayama Study. J Phys Act Health. 2019 Feb 1;16(2):165–171. doi: 10.1123/jpah.2017-0701 Epub 2019 Jan 11. 30634879

[29] Bell S, Lee C. Emerging adulthood and patterns of physical activity among young Australian women. Int J Behav Med. 2005;12(4):227–35. doi: 10.1207/s15327558ijbm1204_3 16262541

[30] Hallal PC, Victora CG, Wells JC, Lima RC. Physical inactivity: prevalence and associated variables in Brazilian adults. Med Sci Sports Exerc. 2003 Nov;35(11):1894–900. doi: 10.1249/01.MSS.0000093615.33774.0E 14600556

[31] Oyeyemi AL, Oyeyemi AY, Jidda ZA, Babagana F. Prevalence of physical activity among adults in a metropolitan Nigerian city: a cross-sectional study. J Epidemiol. 2013;23(3):169–77. doi: 10.2188/jea.je20120116 Epub 2013 Apr 20. 23604060 PMC3700262

[32] Cabinet Office. Economic and Social Research Institute, Tokyo, 2009. [Cited 2021 May 28] Available from: https://www.esri.go.jp/jp/prj/hou/hou050/hou50_03_02.pdf (in Japanese).

[33] Umberson D. Gender, marital status and the social control of health behavior. Soc Sci Med. 1992 Apr;34(8):907–17. doi: 10.1016/0277-9536(92)90259-s 1604380

[34] Eng PM, Kawachi I, Fitzmaurice G, Rimm EB. Effects of marital transitions on changes in dietary and other health behaviours in US male health professionals. J Epidemiol Community Health. 2005 Jan;59(1):56–62. doi: 10.1136/jech.2004.020073 15598728 PMC1763358

[35] Ikeda A, Iso H, Toyoshima H, Fujino Y, Mizoue T, Yoshimura T, et al.; JACC Study Group. Marital status and mortality among Japanese men and women: the Japan Collaborative Cohort Study. BMC Public Health. 2007 May 7;7:73. doi: 10.1186/1471-2458-7-73 17484786 PMC1871578

[36] Jang SN, Kawachi I, Chang J, Boo K, Shin HG, Lee H, et al. Marital status, gender, and depression: analysis of the baseline survey of the Korean Longitudinal Study of Ageing (KLoSA). Soc Sci Med. 2009 Dec;69(11):1608–15. doi: 10.1016/j.socscimed.2009.09.007 Epub 2009 Oct 12. 19819601

[37] Vinther JL, Conklin AI, Wareham NJ, Monsivais P. Marital transitions and associated changes in fruit and vegetable intake: Findings from the population-based prospective EPIC-Norfolk cohort, UK. Soc Sci Med. 2016 May;157:120–6. doi: 10.1016/j.socscimed.2016.04.004 Epub 2016 Apr 6. 27082023 PMC4857700

[38] Cabinet Office. International Comparison Survey of the Daily Life and Attitudes of Elderly Persons in 2010. Tokyo, 2010. [Cited 2021 May 28] Available from: https://www8.cao.go.jp/kourei/ishiki/h22/kiso/gaiyo/pdf/kekka.pdf (in Japanese).

[39] Hawkley LC, Thisted RA, Cacioppo JT. Loneliness predicts reduced physical activity: cross-sectional & longitudinal analyses. Health Psychol. 2009 May;28(3):354–63. doi: 10.1037/a0014400 19450042 PMC2791498

[40] Seino S, Kitamura A, Nishi M, Tomine Y, Tanaka I, Taniguchi Y, et al. Individual- and community-level neighbor relationships and physical activity among older Japanese adults living in a metropolitan area: a cross-sectional multilevel analysis. Int J Behav Nutr Phys Act. 2018 May 25;15(1):46. doi: 10.1186/s12966-018-0679-z 29801456 PMC5970450

[41] Dwyer LA, Patel M, Nebeling LC, Oh AY. Independent Associations and Interactions of Perceived Neighborhood and Psychosocial Constructs on Adults’ Physical Activity. J Phys Act Health. 2018 May 1;15(5):361–368. doi: 10.1123/jpah.2017-0202 Epub 2018 Mar 23. 29569999 PMC5915901

