# Supplementary material for: Association between socioeconomic status and physical inactivity in a general Japanese population: NIPPON DATA2010
Source: PLoS One. 2021 Jul 15;16(7):e0254706. doi: 10.1371/journal.pone.0254706 (PMC8282078; doi:10.1371/journal.pone.0254706)
Supplement: S1 Table — (PDF) [file pone.0254706.s001.pdf]

S1 Table. Distribution of physical activity index by each intensity of activities

	Heavy activity[a]				Moderate activity[b]		Slight activity[c]		Sedentary activity[d]		No activity[e]	
	n	(%)	Median	(IQR)	Median	(IQR)	Median	(IQR)	Median	(IQR)	Median	(IQR)
**Men**												
20-29 years	52	(4.6)	0.0	(0.0, 0.0)	9.0	(0.0, 23.7)	7.1	(4.5, 14.1)	4.7	(3.3, 7.2)	7.3	(6.5, 8.0)
30-39 years	103	(9.1)	0.0	(0.0, 7.5)	4.8	(0.0, 21.6)	8.3	(3.8, 18.0)	3.9	(2.2, 5.5)	7.0	(6.5, 7.5)
40-49 years	122	(10.8)	0.0	(0.0, 10.0)	0.0	(0.0, 20.7)	12.0	(3.0, 18.0)	4.4	(2.8, 6.1)	7.0	(6.0, 8.0)
50-59 years	180	(15.9)	0.0	(0.0, 0.0)	1.2	(0.0, 19.2)	9.0	(4.5, 18.0)	4.4	(3.3, 6.1)	7.0	(6.5, 8.0)
60-69 years	345	(30.9)	0.0	(0.0, 10.0)	3.6	(0.0, 14.4)	6.0	(3.4, 12.0)	6.1	(4.4, 8.8)	8.0	(7.0, 8.5)
70-79 years	252	(22.3)	0.0	(0.0, 5.0)	4.8	(0.0, 12.0)	6.0	(3.8, 8.3)	7.7	(5.0, 9.9)	8.5	(8.0, 9.5)
80-89 years	78	(6.9)	0.0	(0.0, 10.0)	4.8	(0.0, 12.0)	4.5	(3.0, 7.5)	8.8	(5.5, 11.7)	9.0	(8.0, 10.0)
**Women**												
20-29 years	64	(4.3)	0.0	(0.0, 0.0)	12.0	(4.8, 21.6)	6.0	(3.0, 14.3)	5.8	(2.2, 7.7)	7.5	(7.0, 8.0)
30-39 years	221	(15.0)	0.0	(0.0, 0.0)	16.8	(7.8, 24.0)	6.8	(3.0, 12.8)	3.9	(2.2, 6.1)	7.0	(6.5, 8.0)
40-49 years	174	(11.8)	0.0	(0.0, 0.0)	17.4	(9.6, 24.0)	6.8	(3.8, 12.0)	3.9	(2.2, 6.6)	7.0	(6.0, 7.5)
50-59 years	261	(17.7)	0.0	(0.0, 0.0)	14.4	(7.2, 22.8)	6.0	(3.8, 12.0)	4.4	(2.8, 7.7)	7.0	(6.0, 7.5)
60-69 years	389	(26.3)	0.0	(0.0, 0.0)	14.4	(9.6, 20.4)	5.3	(3.0, 8.3)	5.5	(3.3, 7.7)	7.5	(7.0, 8.0)
70-79 years	283	(19.2)	0.0	(0.0, 0.0)	13.2	(7.2, 19.2)	5.3	(3.0, 7.5)	6.1	(4.4, 8.8)	8.0	(7.0, 9.0)
80-89 years	85	(5.8)	0.0	(0.0, 0.0)	8.4	(3.6, 13.8)	5.3	(3.0, 6.8)	7.7	(4.4, 10.2)	9.0	(8.0, 9.5)

IQR; Inter-Quartile Range.

[a]Physical activity index (PAI) of heavy activity was calculated hours for heavy activity multiplied by weighting factor of 5.0.

[b]PAI of moderate activity was calculated hours of moderate activity multiplied by weighting factor of 2.4.

[c]PAI of slight activity was calculated hours of slight activity multiplied by weighting factor of 1.5.

[d]PAI of sedentary activity was calculated hours of sedentary activity (watching television and other sedentary) multiplied by weighting factor of 1.1.

[e]PAI of no activity was calculated hours of no activity (sleeping and lying down) multiplied by weighting factor of 1.0.
