# Supplementary material for: Association between socioeconomic status and physical inactivity in a general Japanese population: NIPPON DATA2010
Source: PLoS One. 2021 Jul 15;16(7):e0254706. doi: 10.1371/journal.pone.0254706 (PMC8282078; doi:10.1371/journal.pone.0254706)
Supplement: S2 Table — (PDF) [file pone.0254706.s002.pdf]

S2 Table. Association between socioeconomic status and physical inactivity

	Men				Women			
	n	%[a]	OR	95% CI	*n*	%[a]	OR	95% CI
**Adult (20-59 years)**								
Employment status								
Working	429	31.9		(ref.)	466	31.3		(ref.)
Not working	28	53.6	**3.35**	**(1.41 − 7.93)**	**254**	**37.0**	**1.44**	**(1.02 − 2.02)**
Educational attainment								
Junior high school	32	28.1		(ref.)	50	42.0		(ref.)
High school	205	26.3	0.92	(0.38 − 2.24)	298	31.9	0.73	(0.38 − 1.43)
College or higher	220	40.5	1.88	(0.76 − 4.61)	372	33.3	0.78	(0.40 − 1.52)
Living status								
Living with spouse	330	33.3		(ref.)	547	30.2		(ref.)
Not living with spouse	127	33.1	0.85	(0.50 − 1.43)	173	43.4	**1.99**	**(1.35 − 2.94)**
Equivalent household expenditure								
1st tertile	167	29.9		(ref.)	205	29.8		(ref.)
2nd tertile	155	32.3	1.16	(0.70 − 1.90)	264	36.7	1.36	(0.90 − 2.05)
3rd tertile	135	38.5	1.37	(0.83 − 2.27)	251	32.7	1.10	(0.72 − 1.67)
**Elderly (60-89 years)**								
Employment status								
Working	304	23.0		(ref.)	177	25.4		(ref.)
Not working	371	40.4	**2.18**	**(1.51 − 3.14)**	580	35.0	**1.69**	**(1.13 − 2.53)**
Educational attainment								
Junior high school	247	31.6		(ref.)	288	32.6		(ref.)
High school	276	31.2	0.99	(0.67 − 1.46)	373	33.1	1.10	(0.78 − 1.54)
College or higher	152	36.8	1.24	(0.78 − 1.94)	96	31.3	1.10	(0.65 − 1.86)
Living status								
Living with spouse	571	30.6		(ref.)	496	30.6		(ref.)
Not living with spouse	104	43.3	**1.65**	**(1.04 − 2.60)**	261	36.8	1.32	(0.92 − 1.88)
Equivalent household expenditure								
1st tertile	193	27.5		(ref.)	248	33.5		(ref.)
2nd tertile	252	35.7	1.43	(0.94 − 2.19)	262	31.3	0.82	(0.56 − 1.21)
3rd tertile	230	33.5	1.28	(0.82 − 2.00)	247	33.6	0.97	(0.65 − 1.43)

OR, odds ratio; CI, confidence intervals. [a]Propotion of defined as participants who physical inactiviy. Physical activity index (PAI) was divided tertile by sex for each 10-year age category and the lowest tertile was defined as physical inactivity. Model was adjusted for all socioeconomic status and confounding factors (age, past histories, alcohol drinking habit, smoking habit, house owner ship, body mass index and living with other), simultaneously.
